# Ferroptosis and pyroptosis in diabetes mellitus: emerging therapeutic potential of GLP-1 receptor agonists

**DOI:** 10.3389/fcdhc.2025.1657710

**Published:** 2025-10-15

**Authors:** Theodoros Panou, Evanthia Gouveri, Manfredi Rizzo, Djordje S. Popovic, Nikolaos Papanas

**Affiliations:** 1 Diabetes Centre-Diabetic Foot Clinic, Second Department of Internal Medicine, Democritus University of Thrace, University Hospital of Alexandroupolis, Alexandroupolis, Greece; 2 Department of Health Promotion, Mother and Child Care, Internal Medicine and Medical Specialties (Promise), School of Medicine, University of Palermo, Palermo, Italy; 3 Clinic for Endocrinology, Diabetes and Metabolic Disorders, Clinical Center of Vojvodina, Novi Sad, Serbia; 4 Medical Faculty, University of Novi Sad, Novi Sad, Serbia

**Keywords:** ferroptosis, pyroptosis, diabetes mellitus, diabetic kidney disease, metabolic-dysfunction associated liver disease, glucagon-like peptide-1 receptor agonists

## Abstract

Ferroptosis and pyroptosis are two emerging forms of regulated cell death. The former encompasses cell death by excessive accumulation of lipid hydroperoxides in an iron-dependent way. The latter pertains to inflammation-associated cell death following activation of caspase-1, caspase-11/4/5 through gasdermin D (GSDMD). Recent evidence confirms the implication of ferroptosis and pyroptosis in diabetes mellitus (DM) and its complications, notably diabetic kidney disease (DKD), and also in metabolic-dysfunction associated liver disease (MASLD). The aim of this narrative review was to summarise current experimental evidence on the potential beneficial actions of glucagon-like peptide-1 receptor agonists (GLP-1RAs) in DM and diabetic complications via reduction of ferroptosis and pyroptosis. Data points to their therapeutic potential in DKD and MASLD. Treatment with GLP-1RAs was comparable with ferrostatin-1 (Fer-1), a well-known-ferroptosis inhibitor: ferroptosis-associated markers (e.g. Acyl-CoA Synthetase Long Chain Family Member 4, ASCL4) were decreased and factors alleviating ferroptosis were increased. Similarly, caspase-1, GSDMD, interleukin-1β (IL-1β) and/or nucleotide-binding oligomerization domain, leucine-rich repeat-containing receptor-containing pyrin domain 3 (NLPR3), which induce pyroptosis, were restored following GLP-1RAs therapy. The pleiotropic effects of GLP-1RAs included improvements in inflammatory markers, fibrosis-associated indices, mitochondrial ultrastructure and oxidative stress. Nevertheless, these positive effects mediated by GLP-1RAs are almost exclusively based on experimental models. Therefore, clinical trials are required to explore these promising outcomes in clinical practice.

## Introduction

Apoptosis has long been considered the only way of regulated cell death and has been characterized by organised cell structure collapse, ranging from the cell membrane to every single organelle (1). Accumulating experimental evidence has pointed to alternative ways of cell death beyond apoptosis, mainly necroptosis, ferroptosis and pyroptosis (2). Particularly ferroptosis and pyroptosis have gathered great interest during the last years due to their role in several conditions, including diabetes mellitus (DM) and diabetes-related complications (3). Clinical evidence supports the efficacy of glucagon-like peptide receptor agonists-1 (GLP-1RAs) in chronic kidney disease (4, 5) or metabolic dysfunction associated liver disease (MASLD) (6). The beneficial effects of GLP-1RAs on renal function are well-established, but their use in MASLD per se is not strongly encouraged (7, 8). However, the impact of novel antidiabetic medications on ferroptosis and pyroptosis has not been extensively studied. Partly the positive effects of GLP-1RAs in clinical studies may be explained through the incretin effect (9, 10). Nevertheless, the overall benefit conveyed by these drugs cannot be fully explained, as not all underlying pathophysiologic mechanisms have been elucidated (11).

Ferroptosis is a novel form of non-apoptotic regulated cell death (12). Since its introduction in 2012, ferroptosis has attracted the interest of researchers across various fields, most notably in cancer research (13). Initially, Erastin, a RAS-selective lethal (RSL) molecule was identified as a ferroptosis-inducer (12). Targets of Erastin, VDAC2 and VDAC3 (VDAC, voltage-dependent anion channel) were localised in the mitochondria, and so it was suggested that Erastin triggered ferroptosis through production of reactive oxygen species (ROS) (12). Erastin was found further to inhibit a heterodimer, composed of glutamate/cystine antiporter solute carrier family 7 member 11 (SLC7A11) and solute carrier family 3 member 2 (SLC3A2), involved in cysteine-dependent glutathione (GSH) synthesis (12). Furthermore, ferrostatin-1 (Fer-1) was proposed as ferroptosis-specific inhibitor which mainly acted as lipid ROS scavenger (12). Additional mediators and inhibitors have also been discussed: glutathione peroxidase 4 (GPX4) suppresses effectively phospholipid hydroperoxides through their reduction; acyl-CoA synthetase long chain family member 4 (ACSL4) and lysophosphatidylcholine acyltransferase 3 (LPCAT3) promote ferroptosis through selectively ligating long chain polyunsaturated fatty acids (e.g. arachidonic, adrenic acid) with coenzyme A (13, 14). Second-line mediators, such as ferroptosis suppressor protein 1 (FSP1), protect cells from ferroptosis upon GPX4 suppression or deletion (15, 16). Further pathways include the 5’ adenosine monophosphate-activated protein kinase (AMPK) pathway and the E-cadherin-neurofibromin 2 (NF2)-Hippo-Yes-associated protein (YAP) pathway, both of which inhibit ferroptosis (17–19). Of note, the impact of glucose in ferroptosis induction is of paramount significance, because erastin cannot promote ferroptosis in the absence of glucose (19). Glucose starvation has been shown to suppress ferroptosis, and so hyperglycemia in DM may induce the exactly opposite effect (13).

Pyroptosis is an alternative form of regulated cell death. Initially, pyroptosis was considered a way for caspase-1 mediated death in response to certain pathogens (20). Nevertheless, the capacity of the recently discovered caspase-11/4/5 in identifying intracellular lipopolysaccharide renders pyroptosis a general non-cell specific type of regulated cell death mediated by innate immunity (20). Gasdermin D (GSDMD) is widely regarded the common substrate for both caspase-1 and caspase-11/4/5 (21, 22). More recent evidence confirms the crucial role of gasdermins. These attach to cell membranes, form pores, impair the osmotic balance of the cell by promoting cell swelling and effectively trigger cell lysis (20). Gasdermins promote secretion of inflammatory cytokines, such as interleukin (IL)-1β and IL-18 (23). Of note, gasdermins are not exclusively activated through inflammasome nucleotide-binding oligomerization domain, leucine-rich repeat-containing receptor-containing pyrin domain 3 (NLPR3) or caspases (20).

In this context, the aim of the present narrative review was to summarise current experimental evidence on the potential beneficial actions of GLP-1RAs in DM and diabetic complications via reduction of ferroptosis and pyroptosis.

## Search strategy

We searched Scopus, PubMed/MEDLINE, and Google Scholar for articles without time restriction, using combinations of the following key words: “ferroptosis”, “pyroptosis”, “diabetes mellitus” and “glucagon-like peptide-1 receptor agonists”. All types of articles (clinical trials, meta-analyses, case-control studies, observational studies, cross-sectional studies, prospective/retrospective studies, cohort studies, comparative studies, randomised studies, experimental studies) were included. Only articles in English were considered.

## Experimental data on ferroptosis

The effect of GLP-1RAs on ferroptosis has been mostly studied in diabetic kidney disease (DKD) and MASLD experimental models. The majority of the experimental studies utilised both an experimental animal mode (mostly db/db mice) and also a selected cell line. Experimental data of GLP-1RAs are presented in **Table 1**.

**Table 1:** Studies on the effect of GLP-1RAs on ferroptosis.

Study	Animal/experimental model and study design	Major outcomes	Conclusions
Song et al. (2022) (24)	db/db mice HepG2 cells	-Diminished glucose, AST and ALT were assessed with liraglutide treatment in db/db mice -Liver fibrosis was ameliorated: fibrotic areas were decreased, collagen I, TGF-β and collagen III levels were insignificantly decreased compared with baseline in db/db mice -Increased expression of SOD, GSH-PX and GSH and decreased expression of MDA, 4-HNE and NOX4 were assessed in db/db mice -Iron deposition was attenuated by reduced expression of TfR1 and increased expression of FPN1 in db/db mice -Significantly increased SLC7A11 expression and NRF2/HO-1/GPX4 pathway activity were documented in liver specimens in db/db mice -Labile iron pools and intracellular lipid reactive oxygen species triggered under high glucose levels were decreased with liraglutide, Fer-1 and DFO -Increased GPX4 and SLC7A11 were found in the western blot analysis in the cells treated with liraglutid	Iron accumulation, oxidative stress and ferroptosis were ameliorated with liraglutide in a T2DM mouse model
Tian et al. (2025) (25)	28 subjects with DKD over a period of 28 weeks -15 subjects in the control arm on insulin -13 subjects on semaglutide STZ-induced diabetic (C57BL/6 mice) model of DKD on high-fat diet HK2 cells	-Significantly improved HbA_1c_, waist-to-hip ratio, serum creatinine and uric acid levels, UACR, NAG, RBF, transferrin were observed among semaglutide users in the clinical substudy -MRI revealed significantly improved renal blood flow, reduced fat accumulation around the kidneys, decreased hypoxia in both the cortex and the medulla, as well as water fractional anisotropy in the clinical substudy -GSH was significantly increased in the group receiving semaglutide, while Fe_2+_, MDA and 4-HNE were reduced in the clinical substudy -In the mouse model, semaglutide increased anti-ferroptosis proteins (SLC7A11, GPX4, FSP1, FTH1, and FPN1) in a similar way as Fer-1, and reduced TfR1 -Semaglutide restored Fe_2+_, MDA and 4-HNE in renal tissue and GSH; NAG and KIM-1 were decreased -The initial histopathologic changes were attenuated by semgalutide and Fer-1 -Inflammatory markers (IL-1β, TNF-α, IL-6, MCP-1 and NF-κB p65 phosphorylation) were decreased and anti-inflammatory markers (such as IL-10) increased with semaglutide; similar results were obtained in HK-2 cells -Semaglutide led to restored levels of TGF-β, Smad2/3 phopshorylation, E-cadherin, α-SMA and vimentin; similar results were obtained in HK-2 cells -In HK2 cells, semaglutide treatment resulted in decreased Fe_2+_ levels, restored TfR1, FPN1 and FTH1 levels and in increased HK-2 cells viability in ferroptosis-imitating conditions (through erastin and RSL3) -Semaglutide treatment resulted in increased mRNA expression of KLB through phosphorylation PKA and CREB) -AMPK/SIRT1/NRF2 pathway mediated the actions of semaglutide in ferroptosis under concomitant high glucose levels; KLB knockdown suppressed the effects of semaglutide on the phsosphorylation of LKB1 and AMPK and subsequent mediators -Similar results were obtained for siRNA experiments resulting in KLB silencing -Similar promising results were observed with liraglutide and dulaglutide	-The beneficial action of semaglutide in DKD was mediated by ferroptosis inhibition
Shen et al. (2024) (26)	STZ-induced diabetic mice Proximal tubular cells	-Exendin-4 administration resulted in significant improvement of ACR, KIM-1 and NGAL in the mouse model -Ferroptosis was significantly improved with exendin-4, as increased expression of ACSL4, reduced expression of GPX4, lipid peroxidation and increased oxidative stress were attenuated in the mouse model; similar outcomes were obtained in proximal tubular cells -Lipid droplets accumulation in the tubule driving ferroptosis was ameliorated with exendin-4 treatment; PLIN2 expression was also improved in proximal tubular cells -Exendin-4 reversed decreased LKB1 and AMPK phosphorylation, elevated SREBP1 and diminished PPARα expression in proximal tubular cells -Restored ATP production and mitochondrial respiration capacity were found in proximal tubular cells -Dorsomorphin counteracted the actions mediated by exendin-4, including the effect on lipid peroxidation in proximal tubular cells -The effects of exendin-4 were also suppressed with the use of Dyngo-4a, a dynamin-dependent endocytosis inhibitor and EIPA, a macropinocytosis inhibitor in proximal tubular cells	-Tubular cell ferroptosis was ameliorated by exendin-4
Deng et al. (2025) (27)	db/db mice treated with dulaglutide db/m mice in the control arm	-Dulaglutide resulted in decreased KLW/BW ratio, serum creatinine and UACR. -Dulaglutide improved histopathological traits -The actions of dulaglutide were mediated by decreased expression of fibrosis-associated genes Col1a2 and Col4a2 and of inflammation-related genes, such as Ccl2, Ccl6 and Cxcl12 -Dulaglutide resulted in significantly decreased ACSL4, SLC7A11, and Ptgs2 expression and insignificantly reduced Chac1 expression -Increased GPX4 expression and decreased ACSL4 expression were present with dulaglutide treatment -Mitochondrial morphology was improved in TEM -4-HNE expression was reduced with dulaglutide -Dulaglutide treatment led to a significant decrease in the expression of several inflammatory markers (IL-6, TNF-α,CCL2, CXCL1, CXCL2, TGF-β, and iNOS) and to decreased F4/80 macrophage infiltration -Both expression of fibrosis-associated genes (Col1a1, Col3a1, Fibronectin, and Acta2) and protein levels of α-SMA, Collagen I, and Fibronectin were restored with dulaglutide -Dulaglutide attenuated increased expression of p-ERK and p-STAT3 and ferroptosis-driven renal cell deat	-Ferroptosis, inflammation and renal fibrosis were reduced with dulaglutide
Chen et al. (2025) (28)	db/db mice db/m mice in the control arm HK-2 cells	-Liraglutide resulted in significant reduction in blood glucose, pyruvate tolerance, HOMA-IR, systolic and diastolic blood pressure, cholesterol, KLW/BW ratio, left kidney weight, creatinine, albumin, BUN levels, β-2 microglobulin and NAG in db/db mice -Renal fibrosis was significantly decreased with liraglutide in db/db mice -Liraglutide resulted in increased expression of T-SOD and CAT and decreased activity of serum 8-OHdG, and decreased MDA, lipid peroxidation, NOX-4,4-HNE and 12-Lox in db/db mice -Liraglutide reversed reduced GSH, GSH-PX, GPX-4 and Fsp1 levels and restored GPX-4 levels in histopathological specimens in db/db mice -Increased ratios of NAD_+_/NADH and CoQ10(H2 )/CoQ10 were also observed upon liraglutide administration in db/db mice -In HK-2 cells exposed to high glucose, liraglutide or Fer-1 administration significantly improved mitochondrial ultrastructure and mitochondrial membrane potential, fluorescence absorption of the lipid perodixation sensor of BODIPY 581/591 C11, restored GPX-4, 4-HNE and Fsp1 expression and the ratios of NAD_+_/NADH and CoQ10(H2)/CoQ10 -Inhibition of Fsp1 through iFsp1 partially obstructed the beneficial actions of liraglutide and Fer-1: reduced GPX-4 and Fsp1 expression and decreased NAD_+_/NADH and CoQ10(H2 )/CoQ10 ratios were observed	-Liragluite attenuated ferroptosis through the Fsp1-CoQ10-NAD(P)H pathway

AST, aspartate transaminase; ALT; alanine transaminase; TGF-β, Transforming Growth Factor-β; SOD, Superoxide dismutase; GSH-PX, Glutathione peroxidase; GSH, glutathione;MDA, malondialdehyde; 4-HNE, 4-hydroxy-2-nonenal; NOX4, nicotinamide adenine dinucleotide phosphate oxidase 4; TfR1, transferrin receptor protein 1; FPN1, ferroportin 1; SLC7A11, solute carrier family 7 member 11; NRF2, nuclear factor erythroid 2-related factor 2; HO-1, heme oxygenase-1; Fer-1, ferrostatin-1; DFO, deferoxamine mesylate; HbA1c, glycated hemoglobin; UACR, urinary albumin/creatinine ratio; NAG, N-acetyl-β-D-glucosaminidase; RBP: retinol binding protein; MRI, magnetic resoncance imaging; FTH1, ferritin heavy chain 1; FSP1, ferroptosis suppressor protein 1; IL, interleukin; TNF, tumour necrosis factor; MCP-1, monocyte chemoattractant protein-1; α-SMA; alpha smooth muscle actin; RSL3, RAS-selective lethal small molecule 3;PKA, protein kinase A; CREB, cyclic adenosine monophosphate -response element-binding protein; AMPK, 5' adenosine monophosphate-activated protein kinase; SIRT1, sirtuin 1; KLB, β-Klotho; LKB1, liver kinase B1; siRNA, small interfering ribonucleic acid; STZ, streptozocin; DM, diabetes mellitus; ACR, albumin/creatinine ratio; KIM-1, Kidney Injury Molecule-1; ACSL-4, Acyl-coenzyme A Synthetase Long Chain Family Member 4; PLIN2, perilipin 2; SREBP1, sterol regulatory element-binding protein 1;PPARα, peroxisome proliferator-activated receptor alpha; ATP, adenosine triphosphate; EIPA, 5-N-ethyl-N-isopropylamiloride; Chac1, ChaC Glutathione Specific Gamma-Glutamylcyclotransferase 1; iNOS, Inducible Nitric Oxide Synthase; ERK, extracellular signal-regulated kinase; STAT, signal transducer and activator of transcription; HOMA-IR, Homeostatic Model Assessment for Insulin Resistance; KLW/BW, kidney weight/body weight; T-SOD, total superoxide dismutase; CAT, catalase; 12-Lox, 12-lipoxygenase; NAD_+_/NADH, nicotinamide adenine dinucleotide oxidised/reduced form ratio; CoQ10(H2)/CoQ10, ubiquinol/ubiquinone ratio; NAD(P)H, Nicotinamide Adenine Dinucleotide (Phosphate); Ptgs2, prostaglandin-endoperoxide synthase 2; CXCR, chemokine (C-X-C motif) ligand; CCL2, C-C motif ligand 2; DKD, diabetic kidney disease.

Tian et al. (25) have included 3 study arms: 13 subjects with DKD on semaglutide and 15 subjects on insulin in the control arm. Furthermore, they used a streptozocin (STZ)-induced mouse model of DKD on high-fat diet and HK2 cells (25). The latter cell line comprises human proximal tubular cells and is usually assessed as an experimental model for DKD (29). The benefit of semaglutide was evident in multiple parameters evaluated, as a significant improvement was found in glycated hemoglobin (HbA_1c_), waist-to-hip ratio, serum creatinine, uric acid, urinary albumin/creatinine ratio (UACR), N-acetyl-β-D-glucosaminidase (NAG), Retinol Binding Protein (RBF) and transferrin (25). Improved renal function was confirmed by magnetic resonance imaging: improved renal blood flow, reduced fat accumulation around the kidneys, decreased hypoxia in both the cortex and the medulla, as well as water fractional anisotropy were common among semaglutide users (25). GSH was significantly elevated in the liraglutide group, while Fe^2+^, malondialdehyde (MDA) and 4-hydroxynonenal (4-HNE) levels were reduced (25). MDA and 4-HNE are markers of lipid peroxidation and routinely assessed in ferroptosis (30). In mice, semaglutide administration markedly enhanced the expression of several anti-ferroptosis proteins, including SLC7A11, GPX4, FSP1, ferritin (FTH1) and ferroportin (FPN1) and also reduced TFR1 levels (25). Of note, these outcomes were also seen in the group receiving Fer-1, regarded the main, yet not exclusive ferroptosis inhibitor (31). Semaglutide treatment reduced increased Fe^2+^, MDA and 4-HNE in renal tissue, and enhanced GSH. NAG and kidney injury molecule 1 (KIM-1) levels were also significantly diminished (25).

Moreover, Tian et al. (25) observed histopathological improvement upon semaglutide administration: the initially assessed deposition of collagen fibers and expansion of mesangial matrix in DKD was ameliorated following either semaglutide or Fer-1 treatment (25). In HK-2 cells treated with semaglutide, decreased Fe^2+^, restored TFR1, FPN1 and FTH1 were observed, along with increased HK-2 cells viability under ferroptosis-imitating conditions (25). The benefit of semaglutide was not restricted to ferroptosis-associated indices. Semaglutide alleviated inflammation in mice and in HK-2 cells: IL-1β, tumour necrosis factor (TNF)-α, IL-6, monocyte chemoattractant protein 1 (MCP-1) and nuclear factor kappa-light-chain-enhancer of activated B cells (NF-κB) p65 phosphorylation were downregulated, while anti-inflammatory markers (such as IL-10) were increased (25). Furthermore, fibrosis-associated markers, such as transforming growth factor (TGF)-β, Smad2/3 phopshorylation, E-cadherin, alpha smooth muscle actin (α-SMA) and vimentin were significantly improved (25). Finally, increased mRNA levels of β-Klotho (KLB) were seen. This was attributed to the phosphorylation of protein kinase A (PKA) and cyclic adenosine monophosphate (cAMP)-response element-binding protein (CREB) (25). Semaglutide exerted its actions also through counteracting oxidative stress in the AMPK/sirtuin 1 (SIRT1)/nuclear factor erythroid 2-related factor 2 (NRF2) pathway (25). KLB knockdown suppressed the effects of semaglutide on the phosphorylation of liver kinase β 1 (LKB1) and AMPK and the subsequent mediators and similar results were observed with the use of small interfering ribonucleic acid (siRNA) promoting KLB silencing (25). Significant results were also obtained with liraglutide and dulaglutide (25). Based on these outcomes, the effect of semaglutide on ferroptosis in DKD is primarily mediated by KLB (25), which is crucial as major anti-aging hormone (32).

Chen et al. (28) examined db/db mice and HK-2 cells treated with liraglutide and heterozygous db/m mice as controls. Liraglutide showed promising results in terms of renal function: it induced significant decreases in blood glucose, gluconeogenesis, improvements in Homeostatic Model Assessment for Insulin Resistance (HOMA-IR), systolic and diastolic blood pressure, serum cholesterol, kidney coefficient (kidney weight/body weight, KLW/BW) ratio, left kidney weight, creatinine, blood urea nitrogen (BUN), β-2 microglobulin, NAG and albumin (28). Renal fibrosis was evaluated through several histopathology stains (e.g. Masson’s staining, Sirius red stain and reticulocyte fiber stain) and through expression patterns of Collagen-I, Collagen-III and TGF-β: it was significantly ameliorated with liraglutide (28). Liraglutide enhanced total superoxide dismutase (T-SOD) and catalase (CAT), and decreased the activity of serum 8-hydroxy-2-deoxyguanosine (8-OHdG), MDA and lipid peroxidation, nicotinamide adenine dinucleotide (phosphate) (NAD(P)H) Oxidase 4 (NOX-4),4-HNE and 12-lipoxygenase (12-Lox), thereby mitigating iron-associated oxidative stress (28). A shift towards increased ratios of nicotinamide adenine dinucleotide oxidised/reduced form ratio (NAD^+^/NADH) and ubiquinol/ubiquinone ratio (CoQ10(H_2_)/CoQ10) was seen (28). In histopathologic specimens, liraglutide improved GSH, GSH-Px, GPX-4 and Fsp1 expression (28). In HK-2 cells exposed to increased glucose, either liraglutide or Fer-1 administration significantly improved mitochondrial ultrastructure and function (28). In particular, these agents improved mitochondrial membrane potential, fluorescence absorption of the lipid perodixation sensor BODIPY 581/591 C11, GPX-4, 4-HNE, Fsp1 and the ratios of NAD^+^/NADH and CoQ10(H_2_)/CoQ10 (28). These benefits were reversed upon inhibition of Fsp1 by iFsp1, suggesting that liraglutide acts through the Fsp1-CoQ10-NAD(P)H pathway (28).

A further experimental study with a similar design assessed the potential of exendin-4 in DKD (26). Exendin-4 administration led to a significant improvement of albumin-creatinine ratio (ACR), KIM-1 and NGAL (26). The effects on ferroptosis-associated indices were also significant: in primary tubular epithelial cells, exendin-4 administration increased GSH and GPX4 (26). Moreover, it reduced expression of ACSL4 (26) The presence of lipid droplets in renal tubule, both in spatial and quantitative terms was significantly attenuated with exendin-4 (26). Expression of perilipin 2 (PLIN2), a lipid droplet associated protein, was improved with exendin-4 (26). Restored LKB1 and AMPK phosphorylation was confirmed with exendin-4 (26). In this study, mitochondrial function optimization was documented through enhanced adenosine triphosphate (ATP) production and respiration capacity, elevated sterol regulatory element-binding protein 1 (SREBP1) and decreased peroxisome proliferator-activated receptor alpha (PPARα) expression (26). These mediators are involved in liver lipid synthesis (33). Simultaneously, glycolysis was ameliorated in tubular cells, thus demonstrating an overall benefit in energy regulation with exendin-4 (26). All these effects were mainly mediated by the AMPK pathway: use of the AMPK inhibitor dorsomorphin counteracted these actions (26). Dyngo-4a, a dynamin-dependent endocytosis inhibitor, and 5-N-ethyl-N-isopropylamiloride (EIPA), a macropinocytosis inhibitor, also suppressed the actions of exendin-4 (26).

Dulaglutide was a further GLP-1RA studied for potentially alleviating renal fibrosis in db/db mice (27). The beneficial effects of dulaglutide on renal function were confirmed, as KLW/BW ratio, serum creatinine levels, UACR and improved histopathological traits were observed (27). These effects were promoted by downregulation of fibrosis-associated genes *Col1a2* and *Col4a2* and of inflammation-related genes, such as *Ccl2, Ccl6*, and *Cxcl12* (27). Dulaglutide administration significantly decreased expression of ACSL4, SLC7A11 and furthermore of a downstream mediator promoting accumulation of lipid peroxides and ferroptosis, prostaglandin-endoperoxide synthase 2 (Ptgs2). It also insignificantly reduced Glutathione Specific Gamma-Glutamylcyclotransferase 1 (Chac1) (27). The latter was found to drive GSH degradation and subsequently cystine-starvation induced ferroptosis (34). GPX4 expression was increased with dulaglutide, contributing to prevention of renal tubular mitochondrial damage (27). The protective role of dulaglutide in mitochondrial morphology was confirmed through transmission electron microscopy (TEM) assessment: it improved decreased density, disrupted cristae and indications of degradation or disappearance (27). Oxidative stress was reduced, as evidenced by diminished 4-HNE (27). Beyond these effects, dulaglutide holds also anti-inflammatory properties (downregulation of IL-6, TNF-α, C-C motif ligand 2 (CCL2), chemokine (C-X-C motif) ligand (CXCL)1, CXCL2), TGF-β, and inducible nitric oxide synthase (iNOS) and diminished F4/80 macrophage infiltration (27). In line with other studies, protein levels of α-SMA, Collagen I, and Fibronectin and the expression of fibrosis-associated genes (Col1a1, Col3a1, Fibronectin, and Acta2) were restored with dulaglutide (27).

A further work assessed the potential utility of liraglutide in alleviating ferroptosis in the liver by studying db/db mice and HepG2 cells (24). HepG2 cells pose a human hepatocellular carcinoma cell line which is widely regarded as a reliable *in vitro* model for MASLD, previously known as NAFLD (35). Liraglutide significantly improved glucose, alanine transaminase (ALT), aspartate transaminase (AST) and liver fibrosis (24). Fibrotic areas decreased (24). Collagen I, TGF-β and collagen III were insignificantly diminished (24). The impact of liraglutide on ferroptosis was significant: on the one hand, there were increased expression of SOD, GSH-PX and GSH, while one the other hand decreased MDA, 4-HNE and NOX4 were seen (24). Iron distribution was also affected, and this was mediated by diminished expression of TfR1 and increased expression of FPN1 (24). Liver sections exhibited increased SLC7A11 expression and NRF2/HO-1/GPX4 pathway activity (24).

## Experimental data on pyroptosis

The effect of GLP-1RAs on pyroptosis has also been studied in MASLD and DKD. Experimental data of GLP-1RAs are presented in **Table 2**.

**Table 2:** Studies on the effect of GLP-1RAs on pyroptosis.

Study	Animal/experimental model and study design	Major outcomes	Conclusions
Yu et al. (2019) (36)	HepG2 cells	-Liraglutide reduced ORO-stained lipid droplets, and this effect was prevented with exendin 9-39 -PA/LPS-induced GSDMD expression and the number of cells stained by PI were found significantly reduced with liraglutide; this effect was inhibited by exendin 9-39 -NLPR3 and caspase-1 p20 expression was decreased with liraglutide; liraglutide ameliorated the expression of NLRP3, caspase-1, caspase-1 p20, and IL-1β; exendin 9–39 prevented the actions of liraglutide -Liraglutide attenuated the reduction in basal respiration and ATP production and the increase in proton leak -Liraglutide maintained respiratory capacity at a higher rate -Liraglutide promoted the presence of functional mitochondria, while exendin 9–39 suppressed this effect -Liraglutide enhanced PINK1-FL and Parkin, Δ2-isoform in cytosol, OPTN and NIX expression, colocalisation of PINK1/Parkin, as well as green-labelled mitochondria; these effects were suppressed by exendin 9-39 -Decreased expression of NLPR3 and GSDMD mediated by liraglutide was counteracted by 3-MA or siRNA against PINK1 pre-treatment	-Liraglutide blocked progression of MASLD through NLPR3-induced pyroptosis inhibition
Shi et al. (2023) (37)	male-specific pathogen-free C57BL/6J mice -control arm -diabetic nephropathy group -diabetic nephropathy group treated with liraglutide -diabetic nephropathy group treated with insulin degludec	-Liraglutide resulted in decreased 24-h urinary protein and total cholesterol in contrast to insulin degludec -Differences in serum creatinine, urea nitrogen and the renal weight/body ratio were insignificant between the different groups -Histopathological renal injury was alleviated with liraglutide, as compared to both insulin degludec and baseline -Liraglutide significantly increased podocyte markers NPHS2 and nephrin in renal immunohistochemical assessment; this contrasted with mice treated with insulin degludec or not receiving any type of treatment -Liraglutide significantly increased GLP-1R expression in glomerular and renal tubular epithelium, as compared to insulin degludec or baseline - Significantly decreased expression of NLRP3, IL-1β, cleaved GSDMD and cleaved caspase-1 was observed following liraglutide treatment -These markers were increased in the diabetic group not receiving treatment or treated with insulin degludec	-Liraglutide exerted its renoprtoective action by counteracting pyroptosis in diabetic nephropathy
Wu et al. (2024) (38)	Male C57BL/6J and ApoE^-/-^mice GEnCs	-Decreased ABCA1 expression was found in GEnCs exposed to high glucose and high cholesterol levels and following DIDS administration -Decreased ABCA1 expression was associated with increased expression of caspase-1, GSDMD and IL-1β, as well as with LDH release and with enhanced cholesterol accumulation in GEnCs -Increased circ8411 expression was observed after exposure to high cholesterol levels, but not after concomitant exposure to high glucose and high cholesterol levels -siRNAs targeting circ8411 resulted in decreased ABCA1 expression, increased expression of caspase-1, GSDMD, IL-1β, increased release of LDH and enhanced cholesterol accumulation -circ8411 knockdown resulted in decreased miR-23a-5p -miR-23a-5p mimic reduced ABCA1 expression, and this effect was blocked by overexpression of circ8411 -RXRα decreased circ8411 and ABCA1 expression -Liraglutide and loxenatide treatment increased ABCA1 expression in GEnCs, reduced cholesterol accumulation along with expression of caspase-1, GSDMD and IL-1β and LDH release; these actions were mediated by increased RXRα and circ84111 expression and by decreased miR-23a-5p expression -siRNA targeting circ8411 suppressed the effects of GLP-1RA on ABCA1 expression -Histological assessment of kidneys from ApoE^-/-^ with DM indicated that liraglutide and loxenatide treatment alleviated renal mesangial hyperplasia and renal tubular vacuolar degeneration -Renal indices (creatinine, BUN and UTP) were decreased with liraglutide and loxeantide treatment, as compared with baseline -In ApoE-/- mice with DM, ABCA1 and circ8411 expression was increased and miR-23a-5p was decreased; these changes were reversed by GLP-1RAs administration -ABCA1 expression was not affected by insulin administration	-GLP-1RAs alleviated pyroptosis through RXRα-circ8411-miR-23a-5- ABCA1 pathway

ORO, Oil Red O; PA, palmitic acid; LPS, lipopolysaccharide; GSDMD, gasdermin D; PI, propidium iodide; NLPR3, nucleotide-binding oligomerization domain, leucine-rich repeat-containing receptor-containing pyrin domain 3; IL, interleukin; PINK1-FL, PINK1 precursor; PINK1, phosphatase and tensin homolog-induced kinase 1; OPTN, optineurin; NIX, NIP3-like protein X; siRNA, small-interfering ribonucleic acid; 3-MA, 3-methyladenine; GLP-1R, glucagon-like peptide-1 receptor; DIDS, 4,4’-diisothiocyanatostilbene-2,2’-disulfonic acid; LDH, lactate dehydrogenase; miR, micro ribonucleic acid; circ8411, circular ribonucleic acid 8411; DM, diabetes mellitus; BUN, blood urea nitrogen; GLP-1RA, glucagon-like peptide-1 receptor agonist; UTP, urinary protein quantityt; GEnCs, glomerular endothelial cells; ABCA1, adenosine triphosphate-binding cassette transporter A; RXRα, retinoid X receptor alpha.

In HepG2 cells, liraglutide reduced oil red o (ORO)-stained lipid droplets (36). As a glucagon-like peptide-1 receptor (GLP-1R) antagonist, exendin 9-39, prevented this decrease in ORO-stained lipid droplets (36, 39). Liraglutide contributed also to increased cell viability (36). The optimal concentration of liraglutide was assessed at 100 nM (36). Palmitic acid (PA) and lipopolysaccharide (LPS), used for MASLD induction, increased GSDMD expression (36). The number of cells stained by PI was significantly reduced with liraglutide, and this was prevented by exendin 9–39 (36). GSDMD is regarded as a major factor leading to a shift from apoptosis to pyroptosis in DKD (40). Liraglutide reduced NLPR3 and caspase-1 p20 expression, and it ameliorated expression of NLRP3, caspase-1, caspase-1 p20 and IL-1β (36). In mitochondria, liraglutide improved ATP production and reduced proton leak (36). Liraglutide enhanced phosphatase and tensin homolog-induced kinase 1 (PINK1)-FL and Parkin, Δ2-isoform in cytosol, optineurin (OPTN) and NIP3-like protein X (NIX) expression, the colocalisation of PINK1/Parkin and the presence of green-labelled mitochondria (36), which are essential against mitochondrial damage (41). The diminished expression of NLPR3 and GSDMD induced by liraglutide was counteracted by 3-methyladenine (3-MA) or siRNA against PINK1 pre-treatment (36).

The impact of liraglutide on pyroptosis and its potential implications in diabetic nephropathy were examined by Shi et al. (37). Overall, 4 groups of male-specific pathogen-free C57BL/6J mice were included: diabetic nephropathy group, diabetic nephropathy group on liraglutide, diabetic nephropathy group on insulin degludec and control group (37). Decreased 24-h urinary protein and total cholesterol was found in mice on liraglutide in contrast to those on insulin degludec (37). Histopathologic evidence pointed to hyperplastic thylakoid stroma and increased glomerular volume, and traits were significantly ameliorated compared to the group on insulin degludec and to baseline (37). Furthermore, liraglutide resulted in significantly higher levels of the podocyte markers NPHS2 and nephrin in renal immunohistochemical assessment (37). This was not seen in mice treated with insulin degludec or untreated mice (37). There was significantly enhanced GLP-1R expression in glomerular and renal tubular epithelium among mice on liraglutide compared with insulin degludec and with baseline (37). Expression of several pyroptosis-related markers, such as NLRP3, IL-1β, cleaved GSDMD and cleaved caspase-1, was subsequently decreased (37).

Wu et al. (38) studied in glomerular endothelial cells (GEnCs) and male C57BL/6J and ApoE^-/-^mice. They treated GEnCs with 1000 nmol/L liraglutide and 80 nmol/L loxenatide, a novel GLP-1RA similar to exenatide with a longer half-time (42). At baseline, decreased ABCA1 expression was found in GEnCs exposed to high glucose and high cholesterol and also following administration of 4,4’-diisothiocyanatostilbene-2,2’-disulfonic acid (DIDS), an ABCA1 inhibitor (38). ABCA1 deficiency has been previously associated with glomerular cholesterol accumulation and dysregulated endothelial function in diabetic kidney disease (43). This was associated with increased expression of caspase-1, GSDMD, IL-1β, enhanced release of lactate dehydrogenase (LDH) and increased cholesterol accumulation (38). Elevated circular ribonucleic acid 8411 (circ8411) expression was documented in the group exposed to high cholesterol, but not under high glucose and cholesterol levels (38). Moreover, siRNAs targeting circ8411 resulted in decreased ABCA1 expression (38). This led to increased expression of caspase-1, GSDMD and IL-1β, increased release of LDH and enhanced cholesterol accumulation (38). There was a protective role of circ8411 against pyroptosis: circ8411 knockdown resulted in increased micro ribonucleic acid (miR)-23a-5p; miR-23a-5p mimic reduced ABCA1 expression, which could be blocked by circ8411 overexpression (38). Liraglutide and loxenatide administration led to increased ABCA1 expression in GEnCs, culminating in decreased cholesterol accumulation, expression of caspase-1, GSDMD, IL-1β and release of LDH (38). These actions were mediated by increased retinoid X receptor alpha (RXRα) and circ84111 expression and by decreased miR-23a-5p expression: circ8411, a circular ribonucleic acid suppressed the effects of GLP-1RA on ABCA1 expression (38). Histological assessment of kidneys from ApoE^-/-^ with DM indicated that liraglutide and loxenatide alleviated renal mesangial hyperplasia and renal tubular vacuolar degeneration (38). In ApoE-/- mice with DM, ABCA1 and circ8411 expression was increased, and miR-23a-5p was decreased. These changes were reversed by GLP-1RA administration (38).

## Discussion

This review has summarised experimental and earliest clinical evidence on the emerging effects of GLP-1RAs on ferroptosis and pyroptosis in diabetes. Mechanisms underlying the therapeutic effect of GLP-1RAs on ferroptosis and pyroptosis are summarised in **Tables 3** and **4**.

**Table 3:** Overview of mechanisms underlying the therapeutic effect of GLP-1RAs on ferroptosis.

Pathway	Condition	Effect on factors promoting ferroptosis	Effect on factors suppressing ferroptosis	Other effects	Reference
KLB/AMPK/ SIRT1/ NRF2 pathway	DKD	Decreased Fe^2+^ and TFR1	Increased GSH, SLC7A11, GPX4, FSP1, FTH1 and FPN1	-Decreased MDA and 4-HNE - Decreased IL-1β, TNF-α, IL-6, MCP-1 and reduced NF-κB p65 phosphorylation -Increased IL-10 -Decreased TGF-β, E-cadherin, α-SMA, vimentin and reduced Smad2/3 phosphorylation,	(25)
Fsp1-CoQ10-NAD(P)H pathway	DKD	N/A	Increased GSH, GSH-Px, GPX4 and Fsp1	-Increased T-SOD and CAT -Decreased 8-OHdG, MDA and lipid peroxidation, NOX-4,4-HNE and 12-Lox - Decreased TGF-β, Collagen-I, Collagen-III -Increased NAD^+^/NADH and CoQ10(H_2_)/CoQ10	(28)
AMPK-fatty acid metabolism pathway	DKD	Decreased ACSL4	Increased GSH and GPX4	-Decreased PLIN2 -Increased LKB1 and AMPK phosphorylation -Decreased SREBP1 and PPARα	(26)
p-ERK and p-STAT3 pathways	DKD	Decreased ASCL4, Ptgs2, Chac1 and SLC7A11	Increased GPX4	-Decreased 4-HNE - Decreased IL-6, TNF-α, CCL2, CXCL1, CXCL2, TGF-β, iNOS and diminished F4/80 macrophage infiltration -Decreased α-SMA, Collagen I, and Fibronectin	(27)
NRF2/HO-1/GPX4 pathway	MASLD	Decreased TfR1	Increased GSH-PX, GSH, SLC7A11 and FPN1	-Decreased Collagen I, Collagem III and TGF-β -Decreased MDA, 4-HNE and NOX4 -Increased SOD	(24)

TGF-β, Transforming Growth Factor-β; SOD, Superoxide dismutase; GSH-PX, Glutathione peroxidase; GSH, glutathione;MDA, malondialdehyde; 4-HNE, 4-hydroxy-2-nonenal; NOX4, nicotinamide adenine dinucleotide phosphate oxidase 4; TfR1, transferrin receptor protein 1; FPN1, ferroportin 1; SLC7A11, solute carrier family 7 member 11; NRF2, nuclear factor erythroid 2-related factor 2; HO-1, heme oxygenase-1; Fer-1, ferrostatin-1; DFO, deferoxamine mesylate; FTH1, ferritin heavy chain 1; FSP1, ferroptosis suppressor protein 1; IL, interleukin; TNF, tumour necrosis factor; MCP-1, monocyte chemoattractant protein-1; α-SMA; alpha smooth muscle actin; RSL3, RAS-selective lethal small molecule 3;PKA, protein kinase A; AMPK, 5' adenosine monophosphate-activated protein kinase; SIRT1, sirtuin 1; KLB, β-Klotho; ACSL-4, Acyl-coenzyme A Synthetase Long Chain Family Member 4; SREBP1, sterol regulatory element-binding protein 1;PPARα, peroxisome proliferator-activated receptor alpha; ATP, adenosine triphosphate; Ptgs2, prostaglandin-endoperoxide synthase 2; Chac1, Glutathione Specific Gamma-Glutamylcyclotransferase 1; iNOS, Inducible Nitric Oxide Synthase; ERK, extracellular signal-regulated kinase; STAT, signal transducer and activator of transcription; T-SOD, total superoxide dismutase; CAT, catalase; 12-Lox, 12-lipoxygenase; NAD^+^/NADH, nicotinamide adenine dinucleotide oxidised/reduced form ratio; CoQ10(H2)/CoQ10, ubiquinol/ubiquinone ratio; NAD(P)H, Nicotinamide Adenine Dinucleotide (Phosphate); CXCR, chemokine (C-X-C motif) ligand; CCL2, C-C motif ligand 2; MASLD, metabolic-dysfunction associated liver disease; DKD, diabetic kidney disease; LKB1, liver kinase B1.

**Table 4:** Overview of mechanisms underlying the therapeutic effect of GLP-1RAs on pyroptosis.

Pathway	Condition	Effect on pyroptosis-associated factors	Other effects	Reference
Mitophagy	MASLD	-Decreased NLPR, IL-1β and caspase-1 p 20 -Decreased GSDMD	-Increased number of MitoGreen (functional) mitochondria -Increased PINK1, Parkin (and their colocalisation), Δ2-isoform in cytosol, OPTN, NIX	(36)
Enhanced GLP-1R expression in kidney	DKD	-Decreased NLPR3, IL-1β and cleaved caspase-1 -Decreased cleaved GSDMD	-Increased NPHS2 and nephrin	(37)
RXRα/circ8411/miR-23a-5p/ABCA1 pathway	DKD	-Decreased caspase-1 and IL-1β -Decreased GSDMD	-Decreased release of LDH	(38)

GSDMD, gasdermin D; NLPR3, nucleotide-binding oligomerisation domain, leucine-rich repeat-containing receptor-containing pyrin domain 3; IL, interleukin; PINK1-FL, PINK1 precursor; PINK1, phosphatase and tensin homolog-induced kinase 1; OPTN, optineurin; NIX, NIP3-like protein X; GLP-1R, glucagon-like peptide-1 receptor; LDH, lactate dehydrogenase; miR, micro ribonucleic acid; circ8411, circular ribonucleic acid 8411; GEnCs, glomerular endothelial cells; ABCA1, adenosine triphosphate-binding cassette transporter A; RXRα, retinoid X receptor alpha; NPSH2, podocin; MASLD, metabolic-dysfunction associated liver disease; DKD, diabetic kidney disease.

Only one study included subjects with DKD (25). All other studies focusing on ferroptosis used exclusively experimental models: HK2 cells (25, 28), HepG2 cells (24) and C57BL6 db/db mice (25, 27, 28). A variety of GLP-1RAs has been used: semaglutide (25), liraglutide (24, 25, 28), dulaglutide (25, 27) and exendin-4 (26). From a methodological perspective, randomisation was present in all studies, and all mice in the experimental arms were treated with fixed drug doses (24–28). This is a notable contrast to the only study encompassing subjects with DKD, as a dose escalation in the insulin detemir (from 0.2 U to 2.0 U) and in the semaglutide (from 0.25 mg to 0.5 mg) groups was noted (25). Mice were mostly 8 to 10 weeks old. Their number varied considerably between 18 and 40 mice across the different studies and dependent on study arms (24–28). Similar conditions were used for cell cultures (24, 25, 28). Ferroptosis was induced in cell cultures with the use of erastin (24), RSL3 (28) and suppressed with the use of Fer-1 (24, 28). Iron chelators (such as deferoxamine mesylate) (24) and other mediators (such as iFsp1) (28) were also applied.

Overall, studies have followed a similar approach. Researchers confirmed that certain commonly used indices in clinical practice (e.g. glucose, UACR) were not within normal range and were restored upon GLP-1RAs treatment (24–28). Some studies also included kidney-specific biomarkers, indicative of renal injury such as NAG (25, 26). Other studies have assessed the effect of GLP-1RAs from an imaging, mainly using magnetic resonance imaging for subjects with DKD (25) and a histopathological perspective through the use of various stains (including hematoxylin and eosin, Masson, Periodic acid-Schiff, Sirius red, reticular fiber stain solution, elastic fiber stain solution, and Perls) (24–28). Researchers also have looked at the effect of GLP-1RAs on ferroptosis-associated markers, oxidative stress and inflammation. Even TEM was applied for the monitoring of therapeutic effects of GLP-1RAs on mitochondrial ultrastructure and beyond (24, 26–28).

The effect of GLP-1RAs on ferroptosis may be seen as promising. Some studies showed a similar effect compared to Fer-1, an established ferroptosis inhibitor, or of deferoxamine (DFO) (25, 28). Ferroptosis-similar conditions were either confirmed through markers or induced through the use of known ferroptosis triggering molecules, such as erastin (25). In all studies, treatment with GLP-1RAs resulted in elevated anti-ferroptosis factors, such as GSH (24–26, 28), GSH peroxidases (24, 28) and particularly GPX4 (24, 26–28), FSP1 (24, 28), FPN1 (24, 25), FTH1 (24, 25) and reduced ferroptosis-associated markers, such as TFR1 (24) or ASCL4 (26, 27).

SLC7A11 has been instrumental in an anti-ferroptosis defense, typically increased upon GLP-1RAs administration (24, 25). Conversely, SLC7A11 was regarded indicative of ferroptosis by a single study and GLP-1RAs treatment led to decreased levels along with other related markers (27). SLC7A11 levels may, in general, vary due to their dependence on other mediators, e.g. GPX4. Nevertheless, this outcome may be attributed to study design, as other discordant results have been reported: decreased GPX4 and increased ASCL4 expressions were found at protein level in db/db mice treated with dulaglutide (27). Researchers themselves attributed the decreased levels of SLC7A11 either to study variability or to previously unrecognised feedback mechanisms (27). These inconsistent findings regarding SLC7A11 render its future use as a marker for evaluating the anti-ferroptosis potential of GLP-1RAs rather unfeasible, although increased levels following treatment with GLP-1RAs should be expected. Furthermore, oxidative stress was significantly ameliorated, as evidenced by the decreased levels of MDA (24, 25, 28), 4-HNE (24, 25, 27, 28), Fe^2+^ (24), NOX-4 (24, 28) and the elevated levels of SOD (24, 28) and CAT (28). Metabolic status of cells improved, mainly due to mitochondria-specific outcomes (increased ratios of NAD^+^/NADH and CoQ10(H_2_)/CoQ10, enhanced ATP production and mitochondrial respiration) (26, 28). Lipid metabolism was also restored, as indicated through restored PLIN2 (26) and diminished lipid peroxidation (26, 28).

Furthermore, GLP-1RAs exhibited both anti-inflammatory and anti-fibrosis properties (e.g. TGF-β), also affecting certain collagen types such as Collagen-I or Collagen-III and respective genes, such as *Col1a2* and *Col4a2* (24, 25, 27, 28). The significance of the AMPK pathway was considered in counteracting oxidative stress (AMPK/SIRT1/NRF2 pathway) and in association with LKB1 (25, 26). The benefit upon GLP-1RAs administration was prevented through AMPK inhibitor dorsomorphin (26). Inhibition of endocytosis and micropinocytosis also inhibited the positive effect of GLP-1RAs (26). The decrease in phosphorylated extracellular signal-regulated kinase (p-ERK) and phosphorylated signal transducer and activator of transcription 3 (p-STAT3) was suggested as a major underlying mechanism responsible for the effect of dulaglutide in renal fibrosis (27). All studies have identified potential signaling pathways mediating the effects of GLP-1RAs on ferroptosis (24–28). Nevertheless, the effect following inhibition of these pathways was examined only in a few of these studies (24, 25, 28). Consequently, not all studies confirmed the suggested mechanisms underlying the effects of GLP-1RAs on ferroptosis.

To date, only a limited number of studies have assessed the effect of GLP-1RAs on pyroptosis in diabetes-associated conditions. Studies on diabetic kidney disease and MASLD have been conducted (36–38). Two cell-based studies including HepG2 and GeNCS and two experimental studies, featuring C57BL/6J mice were conducted (36–38). In these studies, liraglutide was the main GLP-1RA applied (36–38). Furthermore, exendin 9-39 (due to its inhibitory effect) (36), insulin degludec (37) and loxenatide (38) were used. In terms of pyroptosis, liraglutide was associated with diminished GSDMD (36). Liraglutide ameliorated the presence of lipid droplets and enhanced cell viability in the cell model for MASLD (36). In mice, a similar trend towards diminished GSDMD was noted with liraglutide and was associated with restored podocyte markers and improved renal morphology (37). Interestingly, circ8411 was a major protective molecule against pyroptosis (38) and ABCA1 expression was significantly improved with liraglutide (38). Pyroptosis indices, including caspase-1 or IL-1β, were improved with GLP-1RAs (36–38).

Importantly, the cell culture-based study on MASLD examined different liraglutide doses (50 nM, 100 nM, 200 nM and 500 nM) and a fixed exendin 9–39 dose at 100 nM (36). 50 nM exendin 9–39 reversed 100 nM liraglutide and thus this dose was determined as the optimal liraglutide dose for the experiments conducted (36). Due to the particular focus on MASLD, ORO stain was extensively used and TEM was also applied (36). The latter was true for the study including 8-week old C57BL/6J mice randomised to 400 μg/kg liraglutide, 1–2 U insulin degludec and placebo (37). These 24 mice, 8 in each treatment arm, were randomly selected after receiving specific diet and STZ (37). Histopathological assessment was based on hematoxylin and eosin and PAS stains (37). The third available study on pyroptosis encompassed a STZ-induced DKD model with 7 treatment arms: wild-type control group (WT-NC), a diabetic C57BL/6J group, an ApoE-/- group, a diabetic ApoE-/- group and three ApoE-/- groups featuring mice with DM treated with liraglutide, loxenatide or insulin (38). The cell-culture study also used various cell cultures with different conditions: a low glucose control group (5.5 mmol/L), a high glucose group (HG, 25.5 mmol/L), a high cholesterol group (400 μg/ml) and a combined high glucose and high cholesterol group (38). In order to substantiate further the proposed underlying mechanism, inhibitors reversing the therapeutic effects were mostly used, similarly to the studies on ferroptosis (36, 38).

In general, GLP-1RAs hold the most notable therapeutic potential by targeting ferroptosis and pyroptosis. Preliminary outcomes provide an insight into their effect beyond traditional mechanisms of action. Such promising experimental findings pave the way for clinical trials for DKD or MASLD. Metformin has also been studied for its effect in ferroptosis regarding β-cells per se and beyond (44). It has shown an anti-ferroptosis effect in gestational diabetes mellitus and in MASLD models (45–47). The anti-pyroptosis potential of metformin, albeit less studied, is obvious in terms of promising outcomes in diabetic cardiomyopathy through AMPK/mTOR pathway (48). Conversely, the benefit of metformin in MASLD has been attributed to pyroptosis induction, again through the AMPK pathway (49). However, in cancer research, metformin has been used as an agent inducing ferroptosis in various cancer types, such as lung or breast cancer (50, 51). Data on dipeptidyl peptidase-4 inhibitors (DPP-4is) and on sodium-glucose cotransporter-2 inhibitors (SGLT-2is) are extremely scarce (52–55). In a colorectal cancer cell model with tumor suppressor TP53 depletion, several DPP-4is (including vildagliptin, alogliptin and linagliptin) suppressed erastin-induced ferroptosis-mediated cell death, thus pointing to a therapeutic potential beyond DM (52).

Regarding SGLT-2is, the potential utility of dapagliflozin in ameliorating DKD has been demonstrated (53, 54). It was accomplished via ferroptosis suppression by targeting NRF2 and TGF-β signaling pathways or hypoxia inducible factor 1α (HIF1α) and heme oxygenase 1 (HO-1) axis (53, 54). Similar effects have been shown with empagliflozin in DKD through AMPK/NRF2 pathway (55). The utility of DPP-4is in pyroptosis has not gained much interest with the exception of a type 1 diabetes mellitus (T1DM) model which showed that vildalgiptin and linagliptin attenuated lung injury (56). The effect of SGLT-2is in pyroptosis has been assessed in multiple studies, showing some beneficial effects in podocyte pyroptosis in DKD through common pathways (57, 58). Alleviation of skeletal mass loss in a DKD model through dapagliflozin has also been reported (59). Single studies showed promising outcomes in MASLD with canagliflozin and in cardiomyopathy with empagliflozin (60, 61). Nevertheless, the benefit observed in early studies with SGLT-2is is mostly mediated by identical pathways, e.g. in podocytes. Conversely, the potential of GLP-1RAs in both pyroptosis and ferroptosis is based on several and diverse signaling pathways. Consequently, GLP-1RAs have hitherto yielded the most promising results.

To our knowledge, no studies regarding the effect of GLP-1RAs on other forms of cell death, such as necroptosis exist to date (62). The therapeutic potential of targeting mediators implicated in ferroptosis and pyroptosis per se in DM should be also considered: targeting ferroptosis-specific mediators may pave the way for novel therapeutic approaches in DM, e.g. through the selective activation of GPX4 which could further enhance the benefit preliminary observed with therapy based on GLP-1RAs (63). Moreover, the potential effect of GLP-1RAs on other apoptosis-associated molecular mechanisms merits investigation, efferocytosis being a notable example. Efferocytosis, a specialized procedure of apoptotic and necrotic cell clearing has been studied in obesity and the key findings could be further investigated in DM, also in association with ferroptosis and/or pyroptosis (64).

The strengths of this review include comprehensive data analysis and overview of specific mechanisms and mediators in ferroptosis and pyroptosis in DM and diabetic complications. Its major limitations are the limited data available and the heterogeneous study designs, both in the selected models and the various GLP-1RAs used. This heterogeneity prevents generalisation and direct comparisons between study outcomes. Secondly, only a small study included subjects with DKD. Therefore, clinical implementation cannot be at present discussed and needs to be further explored. Early evidence from this single clinical substudy indicated that ferroptosis may partly explain the effects of GLP-1RAs on DKD, pointing to the need for additional clinical research. Large-scale clinical trials could establish the correlations of GLP-1RAs-induced improvements in metabolic parameters of DM and in diabetic complications with markers of ferroptosis and pyroptosis. The potential clinical implications are of great interest, given that these markers might prove useful in monitoring treatment response. Certainly, future basic research and clinical studies should elucidate the potential effects of GLP-1RAs on ferroptosis and/or pyroptosis in other complications, such as diabetic neuropathy. An interesting insight meriting investigation would be the role of GLP-1R multi-agonists in alleviating ferroptosis and/or pyroptosis in DM and beyond. Interestingly, a recent study has reported that retatrutide, a triple agonist of glucagon receptor (GCGR), glucose-dependent insulinotropic polypeptide receptor (GIPR) and GLP-1R, alleviated tumor growth and chemotherapy resistance in obesity-associated triple-negative breast cancer (65). This was achieved by reshaping YAP-proteolytic control, identified as a pathway involved in ferroptosis (17, 65). This experimental study further widens the spectrum of ferroptosis-targeting applications for GLP-1RAs (65). Additional research should now delineate the potential effects of GLP-1R multi-agonists on ferroptosis and pyroptosis, perhaps establishing a more pronounced clinical benefit with such agents.

In conclusion, ferroptosis and pyroptosis represent emerging mechanisms of cell death. Increasing evidence points to their substantial role in DM. GLP-1RAs have yielded promising experimental data in reducing ferroptosis and pyroptosis. Ferroptosis and pyroptosis may emerge as two novel targets for GLP-1RAs. However, clinical experience to confirm or refute these beneficial effects is lacking. Hence, clinical studies are necessary to increase our understanding.
